# African partnerships for patient safety: a catalyst for change in Ethiopia

**DOI:** 10.1186/1753-6561-5-S6-P322

**Published:** 2011-06-29

**Authors:** J Hightower, M Fahmi, M Gashaw, A Derso

**Affiliations:** 1 Patient Safety, World Health Organization, Addis Ababa, Ethiopia; 2 HIV, WHO, Adiss Ababa, Ethiopia; 3 Patient Safety, Gondar University Hospital , Gondar, Ethiopia; 4 Medical Services, Ministry of Health, Addis Ababa, Ethiopia

## Introduction / objectives

African Partnerships for Patient Safety (APPS) stimulated such national actionNational action on infection control in Ethiopia – key steps are outlined.

## Methods

As the result of patient safety sensitization presentations given about the APPS partnership between Gondar Hospital and Leicester Hospital, the Federal Ministry of Health (FMOH) was inspired to begin an extensive pilot program of its own. Technical support was given to the FMOH to clarify specific short and long term patient safety priorities. Existing FMOH infrastructure and partners were identified to provide a platform for training and monitoring & evaluation.

## Results

Patient safety was established as a FMOH priority in the 5 year plan (2010-2015) with specific activities:

1. An Ethiopian FMOH National Patient Safety Program was established prioritizing 4 patient safety action areas in 3 pilot university hospitals. A FMOH technical working group was re-invigorated to cover both patient safety and infection control.

2. FMOH hospital management guidelines revised to include patient safety action areas.

3. National Nursing Standards of Care training material revised to include patient safety.

4. National production of alcohol based hand rub was initiated.

## Conclusion

APPS catalyzed national patient safety action in Ethiopia, resulting in a rapid nationally led integration of patient safety strategy, policy, training, and harmonization of support activities. This potentially sustainable national patient safety program can be replicated elsewhere.

## Disclosure of interest

None declared.

